# Supplementary material for: Diaphragm Ultrasound in Critically Ill Patients on Mechanical Ventilation—Evolving Concepts
Source: Diagnostics (Basel). 2023 Mar 15;13(6):1116. doi: 10.3390/diagnostics13061116 (PMC10046995; doi:10.3390/diagnostics13061116)
Supplement: Supplementary file 1 [file diagnostics-13-01116-s001.zip › diagnostics-2209115-supplementary.pdf]

Table S1 – Reference values of DUS measurements in healthy subjects				
**Author, year**	**n**	**Technique**	**Measure**	**Reference values / Conclusions**
**Harris 1983 (1)**	50	Supine<br>Subcostal<br>MCL and longitudinal orientation	*Mobility<br>- DB	Anterior third: 4.0±1.6 cm<br>Middle and posterior third: 4.8±1.6 cm<br>4.0±1.2 cm (F); 5.4±1.7 cm (M)
**Wait 1989 (2)**	20*	*10 necropsy (the same segment)<br>- US diaphragmatic thickness<br>- Ruler diaphragmatic thickness<br>*10 "in vivo" (sitting) – transducer:<br>- between the ribs, 9th lateral interspace.<br>- End-expiratory thickness (Tdi)	*Thickness-Tdi<br>*TF	- Necropsy (US thickness x ruler thickness):<br>- US diaphragm thickness = 4.4 ± 0.9 mm,<br>- Ruler diaphragm thickness = 4.2 ± 0.9 mm<br>- US and ruler correlation (R = 0.93)<br>- In vivo,<br>- US Tdi = 2.2 ± 0.41 mm.
**Ueki 1995 (3)**	13	Transducer: ZOA<br>- perpendicular to the chest wall<br>- right intercostal space, between AAL-MAL, 0.5-2 cm below the costophrenic angle	*Thickness:<br>- TLC,<br>- FRC,<br>- RV	Mean (SD) thickness:<br>4.5 ±0.9 mm at TLC<br>1.7 ±0.2 mm at FRC<br>1.6 ±0.2 mm at RV
**Gerscovich 2001 (4)**	23	Supine,<br>Longitudinal semi-coronal<br>Subcostal or low intercostal between MCL and MAL	*Mobility<br>- QB	Right hemidiaphragm<br>- QB: 1.5 cm; DB: 5.7 cm; Sniff: 1.7 cm<br>Left hemidiaphragm<br>- QB: 1.6 cm; DB: 6.7 cm; Sniff: 1.8 cm
**Kantarci 2004 (5)**	160	Supine, Coronal plane<br>Low anterior intercostal or subcostal approach, or both	*Mobility<br>- DB	DB = Right: 4.7±1.0 (F) vs 5.3±1.1 cm (M)<br>Left 4.8±0.3 (F) vs 5.4±1.3 cm (F)
**Boussuges 2009 (6)**	210	Standing<br>Right subcostal between MCL-AAL<br>Left lower intercostal spaces or subcostal, between AAL and MAL	*Mobility<br>- QB<br>- DB	QB = -Right: 1.6±0.3 cm (F); 1.8±0.3 cm (M)<br>-Left: 1.6±0.4 cm (F); 1.8±0.4 cm (M)<br>DB = -Right: 5.7±1.0 cm (F); 7.0±1.1 cm (M)<br>-Left: 6.4±1.0 cm (F); 7.5±0.9 cm (M)<br>Sniff= -Right: 2.6±0.5 cm (F); 2.9±0.6 cm (M)<br>-Left: 2.7±0.5 cm (F); 3.1±0.6 cm (M)
**Testa 2011 (7)**	40	Supine, semi-recumbent 45°<br>Subcostal anterior at MCL<br>Probe is orientated transversely and directed cephalad.	*Mobility<br>- QB<br>- DB	QB= - Experienced operator: 1.8±0.8 cm,<br>- Inexperienced operator 2.2±0.9 cm<br>DB= - Experienced operator 6.9±1.4 cm,<br>- Inexperienced operator 7.9±1.3 cm
**Ueki 1995 (3)**	13	Sitting<br>ZOA	*Thickness	Tdi: 1.7±0.2 mm<br>Tdi-max: 4.5±0.9 mm
**Baldwin 2011 (8)**	13	Semi-recumbent 45°<br>ZOA, 9th intercostal space	*Thickness<br>- Tdi	Tdi: 1.7 [1.1–3.0] mm
**Boon 2013 (9)**	150	Supine<br>8th or 9th intercostal space, just anterior to the AAL.	*Thickness<br>-Tdi<br>-Thickening ratio	Tdi: 2.7±1 mm (F); 3.8±1.5 mm (M)<br>- LLN Tdi: 1.4–1.7 mm<br>- LLN Thickening ratio: 1.2 to 1.3 %
**Carrillo-Esper 2016 (10)**	109	Supine<br>ZOA, at FRC	*Thickness<br>- Tdi	Tdi: 1.6±0.4 mm<br>- 1.4±0.3 mm (F); 1.9±0.4 mm(M)
**Cardenas 2018 (11)**	64	Semi-recumbent 45°<br>Mobility:<br>- right anterior, subcostal<br>Diaphragm thickness:<br>- ZOA between AAL and MAL.<br>- at FRC and at TLC.	*Mobility<br>- QB<br>- DB<br>*Thickness<br>- Tdi;<br>- Tdi-max;<br>- TF	Mobility<br>-QB: 1.5±0.4 cm<br>-DB: 6.41±1.02 cm (F); 7.79±0.82 cm (M)<br>-LLN DB mobility: 4.37cm (F); 6.15cm (M)<br>Tdi: 1.9±0.3 mm (M)<br>- LLN at FRC: 1.2 mm (F); 1.3 mm (M)<br>Tdi-max: 4.81±0.95 mm (F); 5.6±0.9 mm (M)<br>TF: 169±43% (F); 204±61% (M)
**Spiesshoefer 2020 (12)**	70	Supine<br>Right hemidiaphragm<br>Excursion: subcostal area.<br>- between MCL and AAL,<br>Thickness: ZOA	*Excursion<br>-QB, DB, sniff.<br>- amplitude<br>- velocity<br>*Thickness:<br>- TLC, FRC	LLNs for diaphragm excursion:<br>- QB: 1.2 cm (M); 1.2 cm (F)<br>- QB Velocity: 0.8 cm/s (M); 0.8 cm/s (F)<br>- Sniff: 2.0 cm (M); 1.5 cm (F)<br>- Sniff Velocity: 6.7 (M) cm/s; 5.2 cm/s (F)<br>- TLC: 7.9 cm (M); 6.4 cm (F)<br>LLN for diaphragm thickness<br>0.17 cm (M); 0.15 cm (F) at FRC<br>0.46 cm (M); 0.35 cm (F) at TLC.
**Boussuges 2021 (13)**	200	Seated<br>Thickness:<br>- ZOA, bilateral<br>-Tdi = thickness at end-expiration<br>- Tmax = after maximal inspiration (deep breathing – DB)	*Thickness:<br>-Tdi<br>-Tmax (DB)<br>-Thickening ratio,<br>- TF	-LLN Tdi: 1.3mm (M) and 1.1mm (F)<br>-TF (M): left side: 60-260%; right side; 57-200%<br>-TF (F): left side: 58-264%; right side: 60-229%<br>- LLN TF (M): right side = 40%; left side = 39%<br>- LLN TF (F): right side = 39 %; left side = 48% (F)<br>- ULN Tmax (DB): 0.78 (F) and 0.79 (M).
**Haaksma 2022 (14)**	46	Supine; Males and Females<br>Probe:<br>- perpendicular to CW,<br>- PAL, MAL and MCL	Thickness:<br>- PAL<br>- MAL<br>- MCL	Thickness:<br>-PAL: M=1.36 [1.23-1.57]; F= 1.08 [0.98-1.24] mm<br>-MAL: M = 1.35 [1.21-1.52]; F =1.15 [1.03-1.28] mm<br>-MCL: M = 2.62 [2.10-2.87]; F =1.99 [1.82-2.37] mm<br>ICC MAL = 0.89, 95% CI: 0.83 - 0.93

		Aim: to assess anatomical variation of thickness on 3 ventrodorsal lines and 2 craniocaudal positions		ICC PAL =0.74, 95% CI: 0.62- 0.85<br>ICC MCL= 0.62, 95% CI: 0.43-0.47<br>* Lowest variability in craniocaudal position = at the midaxillary line (preferred)

Table 2: Reference values of DUS measurements in healthy subjects - adapted form Santana et al, 2020 (15)
MCL: midclavicular line; DB: deep breathing; QB: quiet breathing F: female; M: male; MAL: midaxillary line; TLC: total lung capacity; FRC: functional residual RV: residual volume ; PImáx: maximum inspiratory mouth pressure; TR: thickening ratio; VS: voluntary sniff; AAL: anterior axillary line; ZOA: zone of apposition; Tdi-exp: diaphragm thickness at endexpiration; Tdi-insp: diaphragm thickness at end-inspiration; LLN: lower limit of normal; capacity; TF: thickening fraction; TLC: total lung capacity; PAL: Posterior axillary line; CW: chest wall ; ULN = upper limit for normal

REFERENCES

1. Harris R, Giovannetti M, Kim B. Normal ventilatory movement of the right hemidiaphragm studied by ultrasonography and pneumotachography. Radiology. 1983;146(1):141-4.
2. Wait JL, Nahormek PA, Yost WT, Rochester DP. Diaphragmatic thickness-lung volume relationship in vivo. Journal of Applied Physiology. 1989;67(4):1560-8.
3. Ueki J, De Bruin P, Pride N. In vivo assessment of diaphragm contraction by ultrasound in normal subjects. Thorax. 1995;50(11):1157-61.
4. Gerscovich EO, Cronan M, McGahan JP, Jain K, Jones CD, McDonald C. Ultrasonographic evaluation of diaphragmatic motion. Journal of ultrasound in medicine. 2001;20(6):597-604.
5. Kantarci F, Mihmanli I, Demirel MK, Harmanci K, Akman C, Aydogan F, et al. Normal diaphragmatic motion and the effects of body composition. Journal of ultrasound in medicine. 2004;23(2):255-60.
6. Boussuges A, Gole Y, Blanc P. Diaphragmatic motion studied by m-mode ultrasonography: methods, reproducibility, and normal values. Chest Journal. 2009;135(2):391-400.
7. Testa A, Soldati G, Giannuzzi R, Berardi S, Portale G, Silveri NG. Ultrasound M-mode assessment of diaphragmatic kinetics by anterior transverse scanning in healthy subjects. Ultrasound in medicine & biology. 2011;37(1):44-52.
8. Baldwin CE, Paratz JD, Bersten AD. Diaphragm and peripheral muscle thickness on ultrasound: Intra-rater reliability and variability of a methodology using non-standard recumbent positions. Respirology. 2011;16(7):1136-43.
9. Boon AJ, Harper CJ, Ghahfarokhi LS, Strommen JA, Watson JC, Sorenson EJ. Two-dimensional ultrasound imaging of the diaphragm: quantitative values in normal subjects. Muscle & nerve. 2013;47(6):884-9.
10. Carrillo-Esper R, Pérez-Calatayud ÁA, Arch-Tirado E, Díaz-Carrillo MA, Garrido-Aguirre E, Tapia-Velazco R, et al. Standardization of sonographic diaphragm thickness evaluations in healthy volunteers. Respiratory Care. 2016;61(7):920-4.
11. Cardenas LZ, Santana PV, Caruso P, de Carvalho CRR, de Albuquerque ALP. Diaphragmatic Ultrasound Correlates with Inspiratory Muscle Strength and Pulmonary Function in Healthy Subjects. Ultrasound in Medicine & Biology. 2018.
12. Spiesshoefer J, Herkenrath S, Henke C, Langenbruch L, Schneppe M, Randerath W, et al. Evaluation of respiratory muscle strength and diaphragm ultrasound: normative values, theoretical considerations, and practical recommendations. Respiration. 2020;99(5):369-81.
13. Boussuges A, Rives S, Finance J, Chaumet G, Vallée N, Risso J-J, et al. Ultrasound assessment of diaphragm thickness and thickening: reference values and limits of normality when in a seated position. Frontiers in Medicine. 2021:1982.
14. Haaksma ME, van Tienhoven AJ, Smit JM, Heldeweg ML, Lissenberg-Witte BI, Wennen M, et al. Anatomical variation in diaphragm thickness assessed with ultrasound in healthy volunteers. Ultrasound in Medicine & Biology. 2022;48(9):1833-9.
15. Santana PV, Cardenas LZ, Albuquerque ALPd, Carvalho CRRd, Caruso P. Diaphragmatic ultrasound: a review of its methodological aspects and clinical uses. Jornal Brasileiro de Pneumologia. 2020;46.
